# Recognizing skin conditions in patients with cirrhosis: a narrative review

**DOI:** 10.1080/07853890.2022.2138961

**Published:** 2022-10-29

**Authors:** Ying Liu, Yunyu Zhao, Xu Gao, Jiashu Liu, Fanpu Ji, Yao-Chun Hsu, Zhengxiao Li, Mindie H. Nguyen

**Affiliations:** a Department of Dermatology, The Second Affiliated Hospital of Xi’an Jiaotong University, Xi’an, China; b Department of Infectious Diseases, The Second Affiliated Hospital of Xi’an Jiaotong University, Xi’an, China; c Department of Gastroenterology, The Second Affiliated Hospital of Xi’an Jiaotong University, Xi’an, China; d National & Local Joint Engineering Research Center of Biodiagnosis and Biotherapy, Second Affiliated Hospital of Xi’an Jiaotong University, Xi’an, China; e Center for Liver Diseases, E-Da Hospital, School of Medicine, I-Shou University, Kaohsiung, Taiwan; f Division of Gastroenterology and Hepatology, Fu Jen Catholic University Hospital, New Taipei, Taiwan; g Division of Gastroenterology and Hepatology, Stanford University Medical Center, Palo Alto, CA, USA; h Department of Epidemiology and Population Health, Stanford University, Palo Alto, CA, USA

**Keywords:** Liver cirrhosis, palmar erythema, spider angioma, Terry’s nails, skin manifestations, nail diseases

## Abstract

**Background:** The skin is a major target organ for extrahepatic manifestations of liver diseases, and dermatologic abnormalities are common in patients with hepatic disorders. Clinical examination of the skin, nails and hair can allow for appropriate recognition, early diagnosis and treatment of liver diseases, and improvement in the quality of life and life expectancy of affected patients.

**Methods:** We searched 3 databases (Pubmed,Medline and Embase) and selected studies about cirrhosis related skin manifestations and their pathophysiology.

**Results:** A total of 73 articles were included in the review. Studies displayed the spectrum of cutaneous manifestations related to hormonal and vascular changes as well as nail and hair changes in patients with cirrhosis and/or portal hypertension.

**Conclusion:** Cutaneous alterations are important clues or potential indications in the diagnosis of liver cirrhosis. Familiarity with skin conditions can be promptly diagnosed and appropriate management initiated.

**KEY MESSAGES**
- Manifestations of the liver and skin disorders are interrelated in various ways. Cutaneous changes may be the first clue that a patient has liver disease.
- The skin is a major target organ for extrahepatic manifestations of liver diseases. A broad range of cutaneous alterations can be present in patients with cirrhosis, such as vascular, nail, hair, hormonal changes, etc.
- Recognizing these signs is crucial so that potential underlying diseases including liver disease can be promptly diagnosed and appropriate management timely initiated.

## Introduction

The liver is the second largest organ in the body and is involved in carbohydrate, protein, lipid homeostasis, bile production and storage and filtration of blood [1]. Liver impairment affects almost all body systems, and skin manifestations are common and sometimes the most noticeable aspect of the physical examination and/or symptoms. The majority of cutaneous conditions are asymptomatic requiring no specific therapy, but the diagnosis of cutaneous conditions can often help unmask undiagnosed liver disease and facilitate earlier treatment. Despite modern technological advances, physical examination by a care provider remains the cornerstone of medical diagnosis and management.

Efficient and timely bedside diagnosis of chronic liver disease, especially cirrhosis is particularly relevant, as cirrhosis is the eleventh most common cause of death globally according to a recent report in 2019 [2,3]. In the US, an increase in cirrhosis incidence is also associated with increased mortality, hospitalization and cost in the past decade [4–6].

Therefore, general practitioners as well as hepatology and dermatology specialists should be familiar with cutaneous manifestations of liver disease to facilitate early diagnosis and treatment of liver cirrhosis. In this synopsis, we reviewed the association between hormonal, vascular, nail and hair changes as well as other cutaneous manifestations related to liver disease, focusing on cirrhosis and/or portal hypertension. We will also review the pathophysiologic mechanism underlying these cutaneous conditions in patients with cirrhosis and/or portal hypertension, relevant differential diagnoses (Tables 1–3) and management.

**Table 1.** Dermatologic vascular manifestations associated with liver and non-liver conditions.

Cutaneous condition	Figure	Characteristics of skin lesions	Associated liver conditions	Prevalence	Pathogenesis of cirrhosis related skin conditions	Associated non-liver conditions
Hemodynamic and vascular changes						
Palmar erythema	Figure 1(a)	Blanchable redness in the skin of the thenar eminence and fingertips	Cirrhosis	23%	Changes in peripheral hemodynamics increased free oestrogen levels leading to vasodilation of surface capillaries	Rheumatoid arthritis Hyperthyroidism Diabetes Female of childbearing age
Spider angioma	Figure 1(b,c)	Central arteriole surrounded by capillaries radiating peripherally above the line joining the nipples	Alcohol-associated cirrhosis Hepatopulmonary syndrome	33%		Healthy young adolescent Pregnancy Rheumatoid arthritis Hypertrophic osteoarthropathy
Arteriovenous haemangiomas	Figure 1(d–f)	Bluish erythematous papules or nodules with a diameter of 0.5–1.0 cm in the head and neck	Cirrhosis	NA	Elevated oestrogen levels	Cardiac arteriovenous haemangioma Composite hemangioendothelioma
Caput medusa	Figure 1(h)	Distended veins that radiate from the umbilicus across the abdominal wall resembling a ‘caput medusa’	Cirrhosis Schistosomiasis mansoni Sinus venous thrombosis	NA	Portal hypertension leading to paraumbilical veins that have closed since birth to reopen	Developmental venous anomaly

NA: Data unavailable.

**Table 2.** Nail changes associated with liver and non-liver conditions.

Cutaneous condition	Figure	Characteristics of skin lesions	Associated liver conditions	Prevalence	Pathogenesis of cirrhosis related skin conditions	Associated non-liver conditions
Terry’s nails	Figure 2(a–c)	White nail bed A distal brown to pink transverse band of 0.5–2.0 mm in width Absence of lunulae	Cirrhosis	25.6%	Decrease in vascularity and increase in connective tissue in the nail bed	Congestive heart failure Adult-onset diabetes mellitus Renal failure Human immunodeficiency disease Ageing
Muehrcke’s nail		One or more pale transverse white bands across the nail plate Parallel to the lunula Disappear on pressure	Cirrhosis	NA	Hypoalbuminemia Oedema of the nail bed causing abnormalities in the vasculature of the nail bed	Nephrotic syndrome Chemotherapy Renal insufficiency Cushing’s syndrome
Leukonychia	Figure 2(e)	Diffuse ground glass opacity White in the nail bed	Alcohol-associated cirrhosis	2%	Abnormalities in nail bed vascularization	Hereditary leukonychia totalis Onychopapilloma Psoriasis Chemotherapy HIV infection
Red and blue lunula		Red/blue discolouration of the lunula	Cirrhosis Wilson’s disease	NA	Unknown	Systemic lupus erythematosus Lichen planus Erythonychia Rheumatoid arthritis Alopecia areata Chemotherapy
Clubbing	Figure 2(g)	Increased curvature of the nails Lovibond’s angle Schamroth sign	Cirrhosis Hepatopulmonary syndrome	7%	Elevated platelet-derived growth factor and vascular endothelial growth factor	Cyanotic congenital heart disease Pulmonary fibrosis Inflammatory bowel disease Lung cancer Sarcoidosis HIV infection Tuberculosis Hypersensitivity pneumonitis
Onycholysis	Figure 2(f)	Detachment of the nail bed from the overlying nail plate	Cirrhosis	NA	Hypoalbuminemia	Psoriasis Pemphigus vulgaris Connective tissue diseases Thyroid diseases Hyperthyroidism Diabetes mellitus
Brittle nail	Figure 2(h)	Onychoschizia, onychorrhexis, superficial granulation of keratin and worn-down nail	Chronic liver disease Primary biliary cholangitis	10%	Impairment of peripheral circulation leading to reduced nail matrix vascularization with production of a thin nail plate.	Drug therapy Nutritional deficiencies Psoriasis Pregnancy
Longitudinal striations	Figure 2(i)	Embossed crista on the nail surface accompanied by nail thinning and fracture	Cirrhosis Chronic liver disease	17%	Unknown	Psoriasis Chronic kidney disease Vitiligo
Koilonychia	Figure 2(j)	Concave centrally and raised laterally nail plates	Cirrhosis Hemochromatosis	NA	Nail matrix changes due to blood flow abnormalities	Iron deficiency anaemia Psoriasis Familial koilonychia Trauma
Onychomycosis	Figure 2(k,l)	Nail discolouration, nail separation, brittleness and thickening	Cirrhosis Primary biliary cholangitis	49%	Fungal infection	Trauma Senility Tinea pedis Diabetes Immunosuppression Malignancies Pemphigus vulgaris

NA: Data unavailable.

**Table 3.** Hair, hormonal and other changes associated with liver and non-liver conditions.

Cutaneous condition	Figure	Characteristics of skin lesions	Associated liver conditions	Prevalence	Pathogenesis of cirrhosis related skin conditions	Associated non-liver conditions
Hair						
Loss of pubic hair or beard	Figure 3(a)	Loss of pubic hair	Cirrhosis	NA	Elevated oestrogen level	Trichotillomania Hypothalamic dysfunction XYY-male Sheehan’s syndrome Menopause Treatment with glucocorticoids
Hormonal changes						
Gynaecomastia	Figure 3(b)	Breast enlargement	Cirrhosis	44%	Elevated oestrogen level	Extreme obesity Hypogonadism Adrenal disease Tumours of the adrenal glands, pituitary, lungs and testes Use of digoxin, thiazides, oestrogens, phenothiazines and theophylline Use of methotrexate, alkylating agent, imatinib and vinca alkaloids Use of marijuana Transexual woman Thyroid disorders
Others						
Pruritus			Cholestatic liver diseases Cirrhosis	40-60%	Bile salts, endogenous opioids, serotonin, progesterone metabolites and lysophosphatidic acid	Medications Allergies Atopic dermatitis Psoriasis Kidney disease
Xanthelasma	Figure 3(e)	Pale yellow, planar or slightly bulged, soft plaques around the eyelids	Cholestatic liver disease	NA	Hypercholesterolaemia	Autosomal dominant form of hereditary hypercholesterolaemia Hypercholesterolaemia
Pigmentation	Figure 3(f–h)	Blotchy or diffuse muddy gray coloured hyperpigmentation in the pretibial area	Cirrhosis	46.9%	Red blood cells extravasation and deposition of hemosiderin due to lower extremity edoema and obstruction of venous reflux	Physical dermatosis Inflammatory dermatoses Immunologic dermatoses Allergic/hypersensitivity Cardiac abnormalities
Leg ulcer	Figure 3(i)	Necrotic tissue adhering to basilar part and raised edge	Cirrhosis	NA	Hypoxia-ischemia induced by lower extremity edoema and obstruction of venous reflux	Chronic venous insufficiency Peripheral arterial occlusive disease Vasculitis Diabetes Pyoderma gangrenosum Martorell hypertensive leg ulcer
Coagulation defects		Skin petechiae, ecchymosis, or mucosal bleeding	Cirrhosis	NA	Thrombocytopenia, hyperfibrinolysis, reduction in most factors and inhibitors of clotting and fibrinolytic systems	Haematological diseases Trauma Diabetes Uraemia Anticoagulant therapy for thrombotic diseases

NA: Data unavailable.

## Ethical approval and considerations

This study was carried out in accordance with the International Conference on Good Clinical Practice Standards and the Declaration of Helsinki, and was approved by the Institutional Review Board of The Second Affiliated Hospital of Xi’an Jiaotong University (2018059; Shaanxi, China). The authors obtained written informed consent from all participants in the study. And all participants agreed to publish their de-identified image data.

## Vascular alterations

### Hemodynamic changes and palmar erythema

Liver cirrhosis is characterized by splanchnic and peripheral vasodilation, hyperdynamic circulation and local differences in peripheral circulation between the upper and lower limbs as well as the torso, and these hemodynamic differences predispose to the development of skin vascular abnormalities, including spider veins, palmar erythema and warm hands [7–9].

**Figure 1.**
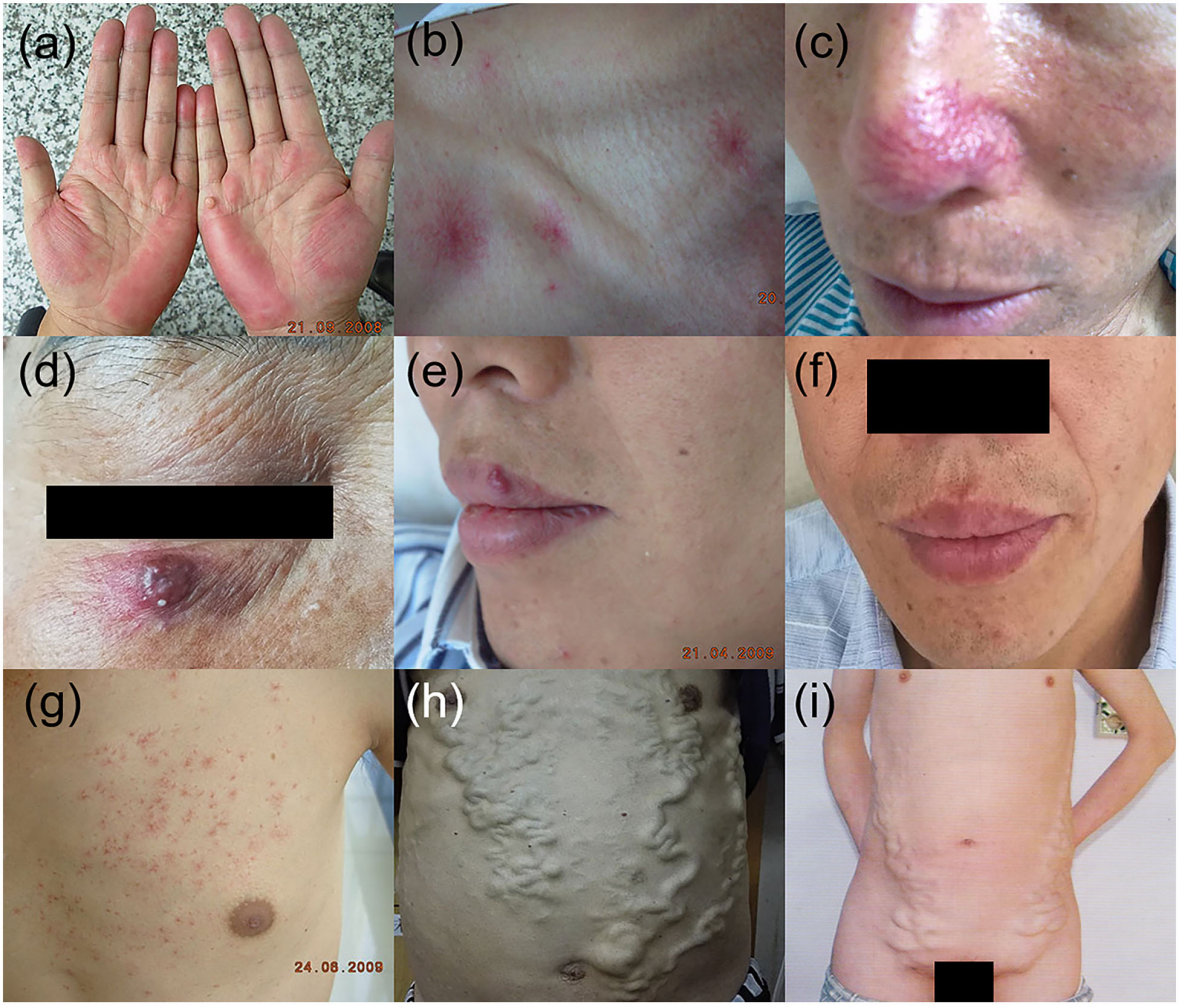
Vascular alterations in patients with chronic liver disease. (a) Palmar erythema, (b, c) Spider angioma, (d) Arteriovenous haemangioma, (e, f) arteriovenous haemangioma on the upper lip of a 38-year-old cirrhotic male with HBV infection, with remission after 10 years of antiviral treatment. (g) Paper money skin, (h) caput medusa, (i) abdominal varicose veins in inferior vena cava obstruction syndrome.

Palmar erythema (Figure 1(a)), also known as liver palms, is characterized by capillary dilatation in the skin of the thenar eminence and fingertips and manifested by blanchable redness of the skin. This condition is caused by elevated serum oestrogen levels and changes in peripheral hemodynamics [8,10]. Though palmar erythema occurs in about two-thirds of patients with liver cirrhosis [10], it is non-specific and can also occur in patients with rheumatoid arthritis, hyperthyroidism, diabetes, juvenile dermatomyositis and pregnancy [10,11].

### Spider angioma

Spider angioma (Figure 1(b)) is a small telangiectatic lesion consisting of a central arteriole from which capillaries radiate peripherally [12]. This entity occurs in 10–15% of healthy individuals, especially adolescents, pregnant women and women who use oestrogen for contraception. However, when multiple spider angiomas appear, they may be a skin manifestation of liver disease, especially alcoholic cirrhosis and hepatopulmonary syndrome [13,14]. Approximately 33% of patients with liver cirrhosis have spider angiomas [15]. Spider angiomas usually range from a few millimetres to a few centimetres in diameter. These lesions are commonly observed in the area of the superior vena cava above the line joining the nipples [13,15]. Spider angiomas localized on the nose (Figure 1(c)) should be differentiated from erythematotelangiectatic rosacea (ETTR). In contrast to painless spider angiomas, ETTR leads to sensitive skin prone to irritation upon contact with skin care products [16].

Younger age as well as increased levels of vascular endothelial growth factor (VEGF) and fibroblast growth factor (FGF) were also found to be independent predictors of spider naevus in patients with liver cirrhosis [17]. The high incidence of spider angiomas in patients with alcoholic liver cirrhosis may occur because ethanol induces VEGF expression and promotes angiogenesis [13,18].

### Arteriovenous haemangioma

Arteriovenous haemangiomas (AVH) (Figure 1(d)) are benign and acquired vascular tumours characterized by bluish erythematous papules or nodules with a diameter of 0.5–1.0 cm. Lesions present as single erythematous plaques in the head and neck in most cases and are more prevalent in middle-aged men [19], which can regress as liver function improves (Figure 1(e,f)). Histopathological features include well-circumscribed dome-shaped lesions in the upper and mid-reticular dermis with dilated thick- and thin-walled endothelium-lined spaces resembling arteries and veins, respectively. In addition, there are also a small number of inflammatory cells infiltrating interstitial cells, while elastic staining indicates the absence of an internal elastic lamina in these vessels, suggesting the dominance of the venous components [20]. The mechanism of AVH is unclear and may be related to elevated oestrogen levels, though Lee et al. found no oestrogen receptors in the biopsy of AVH in patients with liver cirrhosis [19].

### Paper money skin

Paper money skin (Figure 1(g)) is an atypical spider angioma, which manifests as numerous threadlike small blood vessels scattered randomly throughout the skin and disappears under pressure. The distribution is similar to spider angiomas. Lesions are commonly found in the neck, upper torso and upper limbs and resemble security threads on paper money. The mechanism is the same as that of spider angioma [21].

### Caput medusa

Severe portal hypertension of various aetiologies including cirrhosis promotes collateral circulation leading to oesophageal, gastric, abdominal and rectal varices. Abdominal varicose veins can be manifested in the superficial veins of the chest and abdomen presenting a net shape, or abdominal veins with obvious varicose beads or masses. Severe abdominal varicose veins appear as distended veins that radiate from the umbilicus across the abdominal wall resembling a ‘caput medusa’ because the affected veins resemble the snake-like hair of Medusa, a gorgon from Greek mythology (Figure 1(h)) [22].

Caput medusa should be differentiated from vena cava obstruction syndrome (Figure 1(i)). In the latter, abdominal varicose veins are located on the right or bilateral abdomen [23]. The blood flow direction of the abdominal varicose veins of the superior vena cava obstruction syndrome is from top to bottom, and that of the inferior vena cava obstruction syndrome is from bottom to top [24].

Superficial abdominal varicose veins are usually asymptomatic and are found during physical examination or abdominal imaging in patients with advanced liver cirrhosis. However, cases of severe superficial abdominal variceal bleeding have been reported in the literature [25]. Local treatment, such as pressure dressings or sutures, may temporarily control bleeding, but it recurs easily due to unrelieved portal hypertension. Transjugular intrahepatic portosystemic shunt (TIPS) may be effective in treating abdominal variceal haemorrhage and reduces the recurrence rate [26,27].

### Nail changes

Nail abnormalities are also common among patients with liver diseases. Salem et al. found that nail changes occurred in 68% of patients with chronic liver diseases [28]. The most common abnormalities were onychomycosis and longitudinal striations, but several other nail changes are also associated with liver cirrhosis and will be reviewed below. Although non-specific in most cases, nail changes can help provide diagnostic clues to liver cirrhosis.

### Terry’s nails

**Figure 2.**
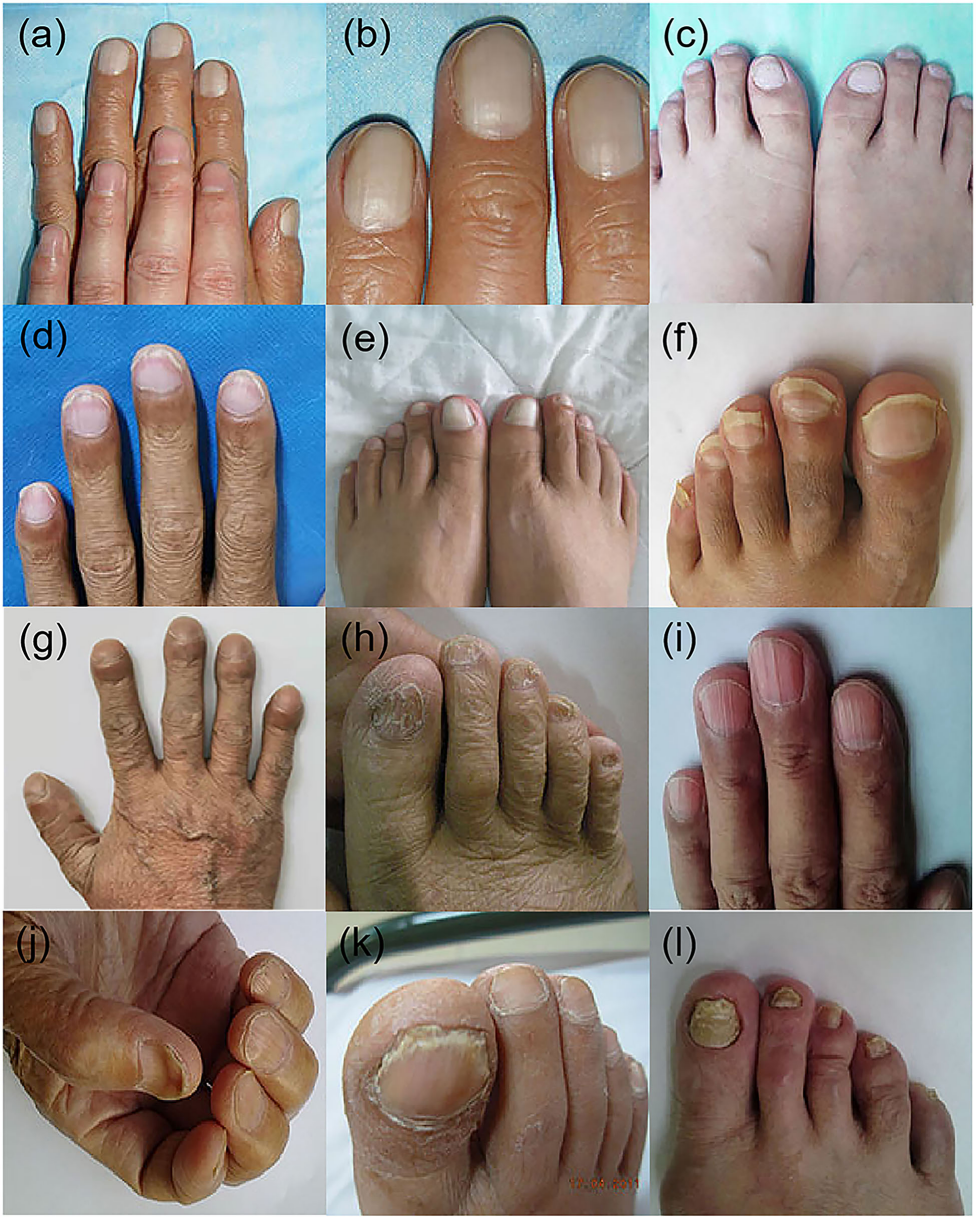
Nail changes in patients with chronic liver disease. (a–c) Terry’s nails, (d) Lindsay’s nails, (e) leukonychia, (g) clubbing nails, (f) onycholysis, (h) brittle nails, (i) longitudinal striations, (j) koilonychia, (k, l) onychomycosis.

Terry’s nails (Figure 2(a–c)) are characterized by the presence of a white nail bed, a distal brown to pink transverse band of 0.5–2.0 mm in width, and the absence of lunulae [29–31]. First reported by Terry in 1954, this condition is a cardinal sign of hepatic cirrhosis [29,32]. However, Holzberg and Walker found a strong correlation between Terry’s nails and congestive heart failure as well as adult-onset diabetes mellitus in addition to cirrhosis in their study of 512 hospitalized patients [33]. Hyperthyroidism, malnutrition, renal failure and Reiter syndrome have also been associated with Terry’s nails [15,34]. In addition, Terry’s nails are associated with ageing in populations without known systemic diseases [33].

The pathogenesis of Terry’s nails is poorly understood but is thought to be related to telangiectasia [33]. Meanwhile, Lindsay’s nails (Figure 2(d)), the most common differential diagnosis of Terry’s nails, are characterized by the distal extension of the lunula and a pink or reddish-brown distal transverse band, and this condition is closely linked to chronic renal diseases [35].

### Muehrcke’s nail

Muehrcke’s nail is a type of leukonychia in which the second, third and fourth fingernails have one or more pale transverse white bands across the nail plate, parallel to the lunula, which disappear on pressure [36]. This condition indicates hypoalbuminemia secondary to cirrhosis, nephrotic syndrome, pellagra and sprue and can resolve by increasing serum levels of albumin [37]. Muehrcke’s lines constitute an abnormality of the vascular nail bed that does not shift as the nails grow [38]. This abnormality is also associated with chemotherapy [39,40].

### Leukonychia

Leukonychia (Figure 2(e)), or whitening of the nail plate, also known as ongchostoma sima, is a common disease that was first described in 1919. Leukonychia is classified as acquired or congenital and may be due to an abnormality of the nail bed (pseudoleukonychia) or nail plate (true leukonychia) [41]. True leukonychia showed diffuse ground glass opacity and white colour of the nail bed, which was different from Terry’s nail in that there was no distal pink or brown band.

### Red and blue lunula

Red lunula refers to the blanchable red discolouration of the lunula and occurs in patients with cirrhosis, cardiac failure, systemic lupus erythematosus, chronic urticaria, psoriasis and carbon monoxide poisoning [42]. The azure lunula is the blue discolouration of the lunula that occurs in Wilson’s disease, a hereditary disorder of copper metabolism [43].

### Clubbing and hypertrophic osteoarthropathy

Clubbing (Figure 2(g)) is the incrassation of the soft organization beneath the proximal nail plate, leading to increased curvature of the nails. Diagnostic findings include Lovibond’s angle, and the Schamroth sign [44]. This condition may indicate cyanotic congenital heart disease, pulmonary fibrosis, bronchial carcinoma, inflammatory bowel disease, cirrhosis and thyroid acropachy. Although the underlying mechanism remains elusive, several hypotheses have been proposed, including neurocirculatory reflex, growth hormone and megakaryocyte/platelet clump [45]. It is important to differentiate between clubbing and hypertrophic osteoarthropathy, which may resemble clubbing but is distinguished from clubbing by the presence of a painful nail bed, while clubbing is asymptomatic. Hypertrophic osteoarthropathy is associated with the paraneoplastic syndrome of several malignancies, including primary liver cancer [46].

### Onycholysis

Onycholysis (Figure 2(f)) is the detachment of the nail bed from the overlying nail plate and may be due to trauma, manicuring and photodermatitis. While psoriasis is the most prevalent disease leading to onycholysis [47], there is also a significant association between liver cirrhosis and onycholysis, which have been reported to resolution following liver transplant [48].

### Brittle nail syndrome

Brittle nail syndrome (Figure 2(h)) is characterized by increased fragility of the nail plate. The main clinical features are onychoschizia, onychorrhexis, superficial granulation of keratin and worn-down nail. This condition affects up to 20% of the population, especially women over 50 years of age. Brittle nails can be either inherited or acquired and are associated with systemic diseases including liver cirrhosis, drug therapy, nutritional deficiencies, trauma, infections and nail dehydration [49,50].

### Longitudinal striations

Longitudinal striations (Figure 2(i)) are embossed crista on the nail surface, accompanied by nail thinning and fracture. The presence of longitudinal striations is a common form of nail dystrophy, which is caused by a deficiency in vitamins and calcium. The condition is termed 20-nail dystrophy in cases involving all nails [40,51].

### Koilonychia

Koilonychia (Figure 2(j)) (spoon-shaped nails) is a disorder in which nail plates are concave centrally and raised laterally. Water droplets can converge on the hollow nail plate and can serve as a useful diagnostic tool [52]. Chinazzo et al. performed a prospective observational study and found that the prevalence of koilonychia in healthy newborns was 32.7% [53]. While the abnormality is a natural variant, it is associated with iron deficiency anaemia, hemochromatosis, coronary disease, hypothyroidism and cirrhosis, giving diagnostic clues to these conditions when present in an appropriate context [43].

### Onychomycosis

Onychomycosis (Figure 2(k,l)) is a common nail disease caused by dermatophytes, nondermatophytes and yeast. Clinical manifestations include nail discolouration, nail separation, brittleness and thickening that may worsen over time. It may cause local pain, paresthaesia, difficulty performing activities of daily living, and social isolation. Predisposing factors include trauma, senility, tinea pedis, diabetes, immunosuppression and cirrhosis [54,55]. The prevalence of onychomycosis in primary biliary cholangitis (PBC) patients is as high as 49% [55,56].

### Hair and hormonal changes

**Figure 3.**
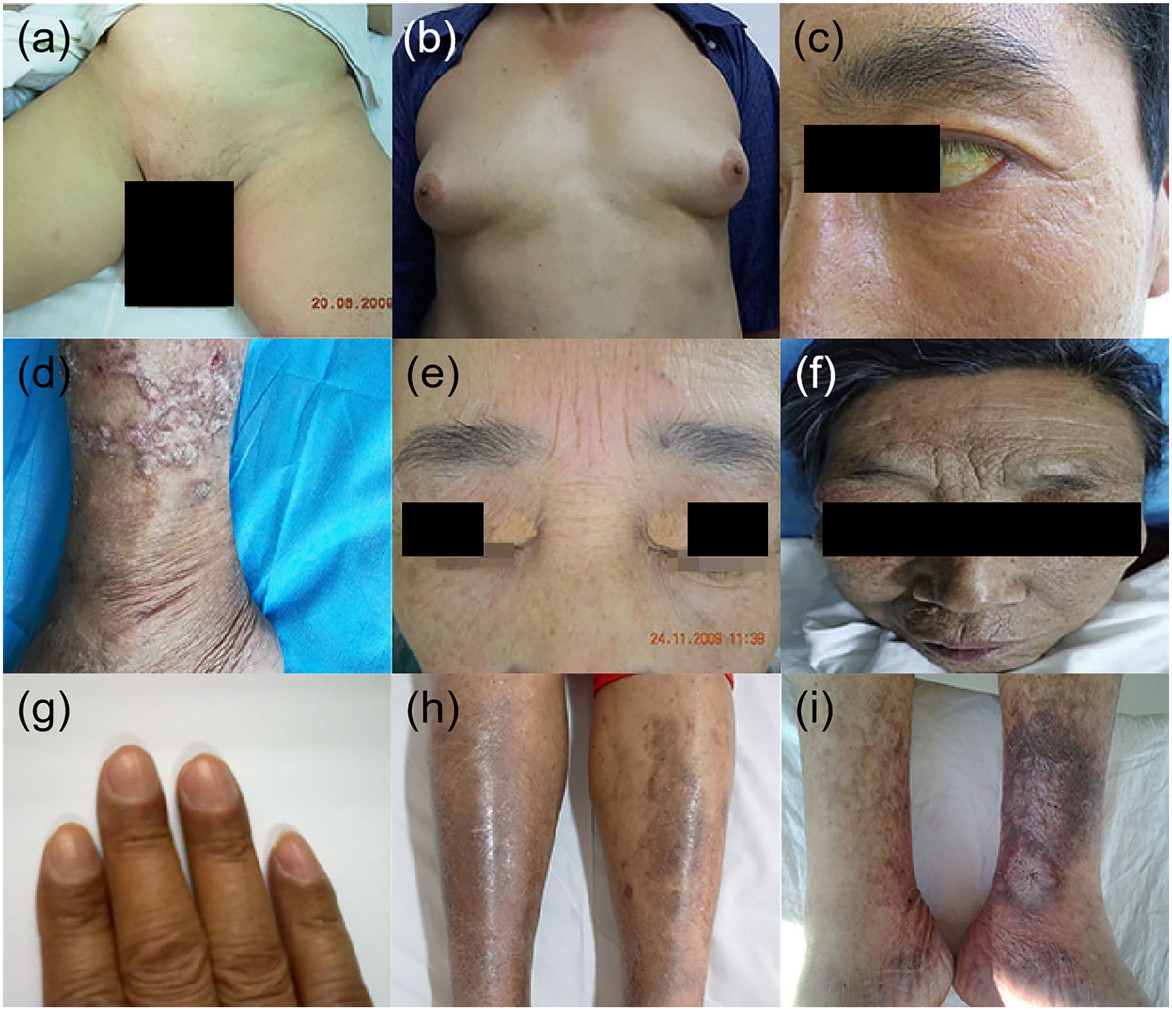
Hair, hormonal and other dermatologic manifestations in patients with chronic liver disease. (a) Loss of pubic hair, (b) gynaecomastia, (c) jaundice, (d) prurigo nodularis, (e) xanthelasmas, (f–h) pigmentation, (i) leg ulcer.

Chronic liver diseases, especially end-stage liver disease such as decompensated cirrhosis, are associated with loss of axillary, arm and pubic hair (Figure 3(a)). In men, liver cirrhosis is linked with a decrease in the growth rate of facial hair, female pubic hair pattern, loss of libido, testicular atrophy, oligospermia and gynaecomastia (Figure 3(b)). Gynaecomastia is the first symptom of liver cirrhosis in some cases and should be differentiated from the gynaecomastia induced by hormone therapy for prostate cancer [57,58]. Commonly-used medications in patients with cirrhosis and ascites such as spironolactone can exacerbate symptoms of gynaecomastia and an alternate dose or therapy may be required [59].

## Other dermatologic manifestations

### Jaundice

Jaundice (Figure 3(c)) describes discolouration of the skin, sclera and mucous membranes that is attributable to the accumulation of bilirubin and its metabolites in the tissues. Jaundice generally begins to become visible when the concentration of serum bilirubin surpasses about 2 mg/dL (34 mmol/L). The colour of the skin varies from lemon yellow to apple green, gradually evolving as the serum bilirubin level becomes elevated. According to the presence of conjugated or unconjugated components of bilirubin, we can classify the three major groups of underlying causes of jaundice: prehepatic, intrahepatic or post-hepatic. Prehepatic jaundice involves haemolytic anemias, which can also be seen in neonatal physiological jaundice and breast milk jaundice. Intrahepatic jaundice involves both conjugated and unconjugated hyperbilirubinemia resulting from liver failure-associated severe acute hepatitis or cirrhosis. Other intrahepatic causes of hyperbilirubinemia include intrahepatic cholestatic diseases, such as PBC and the various congenital genetic disorders involving bilirubin metabolism or transport such as Gilbert, Crigler–Najjar syndrome, or Dubin-Johnson syndrome. Post-hepatic jaundice involves conjugated hyperbilirubinemia arising from extrahepatic issues, such as biliary tract obstruction [1].

### Pruritus

Pruritus is a sensation that induces persistent or intermittent itching and involuntary scratching. It can affect the whole body or be confined to the limbs, especially the footplate and palm, where more intensive itchiness may occur. It is one of the most common skin abnormalities that occur in liver disease, particularly in patients with cholestatic liver disease. As a frequent concomitant symptom without visible lesions of liver cirrhosis, pruritus is usually linked to cholestasis in PBC, primary sclerosing cholangitis, obstructive gallstone disease and carcinoma of the bile duct. It can also be the most prominent symptom in certain pregnancy-associated liver conditions such as intrahepatic cholestasis of pregnancy [60]. Viral hepatitis-related cirrhosis can lead to intense pruritus, accompanied by solid crusty nodules, which are called prurigo nodularis (Figure 3(d)). Usually distributed in the extremities, especially between the knee and ankle and forearm, the lesion is associated with the topical deposition of an immune complex consisting of HBV/HCV in the skin [1]. Data from a large cohort of patients with chronic liver disease (*n* = 1631) suggest that the overall prevalence of pruritus was about 40% overall, higher among those with cirrhosis and as high as 50% in those with PBC and 60% in those with autoimmune overlap syndrome [61].

Pruritus may be intermittent and minimally symptomatic but when persistent can lead to a dramatic reduction in quality of life, insomnia, depression and even suicidal attempts. The intensity or pruritus appears to follow a nyctohemeral rhythm, being most severe in the late evening [62,63]. While the health-related quality of life and severity of pruritus appears to be exacerbated by the presence of advanced diseases such as cirrhosis, the ailment development in PBC is not linked to the severity of pruritus [64,65].

The pathogenesis of pruritus in cholestasis is complicated and obscure. Bile salts, endogenous opioids, serotonin, progesterone metabolites and lysophosphatidic acid have all been considered contributory factors [66]. However, the precise role of this substance is unclear due to the lack of an observed relation between concentrations of these molecules and the intensity of the pruritus. Lysophosphatidic acid (commonly referred to as LPA), produced by the enzyme autotaxin (ATX), has also been characterized as an underlying pruritogen in cholestasis. ATX activity and serum LPA levels correlate with the severity of pruritus, suggesting the potential role of each as therapeutic target [67].

### Xanthelasmas or xanthomas

Xanthelasma (Figure 3(e)) manifests as pale yellow, planar or slightly bulged, soft plaques around the eyelids, being essentially subcutaneous lipid deposits. The condition is associated with dyslipoproteinaemia secondary to liver diseases such as PBC and other forms of cholestatic liver disease. The condition is seen most frequently in females over 50 years old, and half of the cases present with comorbid dyslipidemia. Xanthelasmas associated with the autosomal dominant form of hereditary hypercholesterolaemia develop during childhood and the clinical profile includes xanthelasma, tendon xanthoma, increased low-density lipoprotein, arcus corneae and premature coronary artery disease [68]. One case of regression has been reported after liver transplantation in a patient with PBC [69].

### Pigmentation

Patients with cirrhosis usually have abnormal pigmentation. The pigmentation on the face is manifested by a muddy gray complexion, called hepatic face (Figure 3(f)), which is induced by hormone metabolism turbulence. Pigmentation can also occur in terminals of the extremities (Figure 3(g)), presenting with black discolouration, especially common in patients with cirrhosis. When occurring in the tibial anterior (Figure 3(h)), pigmentation is caused by the extravasation of red blood cells and deposition of hemosiderin due to lower extremity edoema and obstruction of venous reflux [70].

### Leg ulcer

Leg ulcer (Figure 3(i)) appears as a lesion in which necrotic tissue adheres to the basilar part and the edge is raised, often occurring on the tibial anterior. It is related to hypoxia-ischemia induced by lower extremity edoema and obstruction of venous reflux.

### Coagulation defects

Cirrhosis is considered to be a thrombosis-prone state because venous stasis from portal hypertension and the inflammatory milieu promote the development of portal vein thrombosis [71]. However, coagulation defects can also be observed in cirrhosis [72,73]. In patients with cirrhosis, coagulation defects manifest as skin petechiae, ecchymosis, or mucosal bleeding, the causes being complex and multiple. Cirrhosis and portal hypertension are often marked with thrombocytopenia. While patients with cirrhosis typically have platelet counts adequate for the production of thrombin at a level equivalent to the rock bottom of the normal extent in a healthy population, the balance of fibrinolysis may be altered by acute events such as infection leading to hyperfibrinolysis, which increases the risk of haemorrhage. Moreover, in patients with cirrhosis, protein synthesis often is diminished, leading to a dramatic reduction in most factors and inhibitors of clotting and fibrinolytic systems, leading to further coagulation defects [72,73].

## Conclusions

A broad range of cutaneous alterations can be present in patients with liver cirrhosis, portal hypertension and other chronic liver diseases. Vascular changes in the upper part of the body are important clues in the diagnosis of liver cirrhosis, and toenail changes and other skin signs may indicate potential chronic liver diseases, especially liver cirrhosis. While the majority of these cutaneous conditions are asymptomatic, some may require specific therapy, such as pruritus and all should be recognized as signs of potential underlying diseases, including liver cirrhosis, so that underlying conditions can be promptly diagnosed and appropriate management initiated.

## Author contributions

Conception and design: Y.L., Y.Z., F.J., Z.L. and M.H.N.; literature search and investigation: Y.L., Y.Z., X.G. and L.J.; writing-original draft: Y.L., Y.Z. and F.J.; writing-review and editing: F.J., Y.H., Z.L. and M.H.N.

## Data Availability

The datasets used and/or analyzed during the current study are available from the corresponding author on reasonable request.
